# Launch meeting of the new World Health Organization Collaborating Centre for Antimicrobial Resistance, Consumption and Health Care-Associated Infections at the Robert Koch institute, Berlin, Germany, October 2022

**DOI:** 10.2807/1560-7917.ES.2022.27.47.2200895

**Published:** 2022-11-24

**Authors:** RKI Unit on HAIs and Surveillance of AMR and AMC - International Team, RKI Unit on HAIs and Surveillance of AMR and AMC - International Team, Sara Tomczyk, Arina Zanuzdana, Annika Heck, Sebastian Haller, Muna Abu Sin, Tim Eckmanns

**Affiliations:** 1 Unit 37 Healthcare-associated Infections, Surveillance of Antibiotic Resistance and Consumption, Department of Infectious Disease Epidemiology, Robert Koch Institute, Berlin, Germany; 2 Members of the RKI Unit on HAIs and Surveillance of AMR and AMC - International Team are listed under Collaborators

**Keywords:** AMR, IPC, WHO, Collaborating Centre, Surveillance, Network

On 18 October 2022, the German national public health institute, Robert Koch Institute (RKI), convened a meeting to launch a new World Health Organization (WHO) Collaborating Centre (CC) for AMR, Antimicrobial Consumption (AMC) and Health Care-Associated Infections (HAI) and to bring together international experts for a panel discussion on the critical global gaps and existing opportunities to leverage for improved AMR prevention and control.

In 2019, an estimated 4.95 million deaths were associated with bacterial antimicrobial resistance (AMR), making it one of the leading causes of death worldwide [1]. Such data are critical for policy development on infection prevention and control (IPC) and patient management, including access to diagnostics and treatment. They underscore the need for global collaboration and awareness to continue to address important gaps in AMR prevention and control.

The World Health Organization (WHO) coordinates a network consisting of more than 25 collaborating centres (CC) from 17 countries across the globe, and since 2019 RKI supports the network coordination [2]. In particular, the network supports low- and middle-income countries to build capacity for AMR surveillance. Therefore, the AMR Surveillance CC Network comprises a broad set of stakeholders with AMR expertise in human, animal and environmental health and with laboratory and epidemiology capacities [3].

The WHO CC AMR launch event was held following the World Health Summit (WHS) in Berlin, Germany, in the same week, during which political leaders and speakers stressed the fundamental need to “act together and effectively to ensure a healthy life for all on a healthy planet.” The WHS included a session on AMR as a global challenge and the task of national public health institutes in combatting its threat [4]. In this session, the panellists explained how the AMR pandemic sets us further back from achieving universal health coverage and the United Nations Sustainable Development Goals (SDGs). The WHO CC AMR launch event aimed to build upon this momentum and to further focus the discussion on critical global gaps and existing public health opportunities in the ongoing AMR pandemic.

The event was opened by Sara Tomczyk (Unit on HAIs and Surveillance of AMR and AMC, RKI) and welcome remarks were given by Carmem Pessoa-Silva (AMR Surveillance Unit, WHO, Geneva, Switzerland), Benedetta Allegranzi (Infection Prevention and Control Hub, WHO, Geneva, Switzerland), and Dagmar Reitenbach (Department of Global Health Policy at the German Federal Ministry of Health, Berlin, Germany). Under the moderation of Johanna Hanefeld (Centre for International Health Protection, RKI), a panel discussion was then held with Hanan H. Balkhy (AMR, WHO), Ruth Schumacher (German Development Agency (GIZ), Berlin, Germany), Sabiha Essack (University of KwaZulu-Natal, South Africa) and Muna Abu Sin (Unit on HAIs and Surveillance of AMR, RKI). Both in-person and digital participation from the broader international community working on AMR prevention and control was offered.

Throughout the remarks and discussions, five key themes emerged including the importance of: (i) a multisectoral approach with societal and political engagement; (ii) a patient-centred focus; (iii) universal health coverage for diagnostics, treatment and infection prevention and control (IPC); (iv) strengthened antimicrobial stewardship; and (v) an effective role of national public health institutes and surveillance systems.

## Multisectoral approach with societal and political engagement

The coronavirus disease (COVID-19) pandemic exposed the gaps and dysfunctionalities in global health governance and local systems. Speakers underlined the need for actors across sectors to leverage these lessons and work together in more meaningful and actionable ways on the prevention and control of AMR. It was agreed that this should include initiatives to improve health literacy on AMR and make the economic case for its prevention and control. Policymakers, practitioners and civil society should work together to develop consistent, accurate and tailored messages to encourage societal and political ownership on preventing and controlling AMR, taking local circumstances into account. Examples given included: (i) building on the ‘lived experience’ of the health, social, economic and political impacts of the COVID-19 pandemic but framing AMR as a One Health issue with even more far-reaching impacts as demonstrated by data such as the recent global AMR burden study [1]; (ii) emphasising the need for a ‘whole of society’ approach and government response including both top-down and bottom-up mobilisation for investments in surveillance, stewardship and IPC and highlighting the costs of inaction; and (iii) inclusion of AMR as a central part of global multi-sectoral initiatives and instruments such as the pandemic treaty proposed by the Global Leaders Group on AMR.

## Patient-centred focus

As it relates to healthcare service delivery and public health measures for the prevention and control of AMR, speakers agreed that the individual patient needs to be more explicitly at the centre of planning and implementation. AMR prevention and control measures should be patient- or people-centred with a need for equitable access to diagnostics, treatment and immunisation. This could also include collaboration with patient organisations and consideration of patient needs in activities such as AMR, AMC and HAI surveillance and data collection which can lead to better patient outcomes. WHO is currently working on implementing a people-centred approach to address AMR by supporting countries with national action plans, technical programmes, advocacy, community engagement and further efforts. An urgent need for support of local and national clinical networks as well as support of a global microbiological laboratory network to improve diagnostics was emphasised in this context. Improving diagnostics for patients with timely results that allow for improved patient management and surveillance quality should be among the first steps.

## Universal health coverage for diagnostics, treatment and infection prevention and control

There was common acknowledgement among speakers that affordable procurement of diagnostics and antimicrobial therapy and reliable supply chains continues to be a notable challenge in low- and middle-income countries, hindering improved AMR prevention and control. Long-term support of development of diagnostic capacities which would feed the surveillance data in those countries is needed. Diagnostic material such as blood culture bottles remain expensive, and in many countries, patients must pay for diagnostics out of pocket. These issues should be prominently included on universal health coverage agendas, and advocacy and mitigation could include ideas such as intensified public-private engagement and leveraging of combined purchasing power of countries for better access to diagnostic and antimicrobial products among other ideas.

## Strengthened antimicrobial stewardship

Speakers also highlighted the importance of strengthening antimicrobial stewardship efforts. Globally, this necessitates balancing three important aspects: (i) equitable access to antimicrobial therapy in the context of universal health coverage; (ii) conservation to make the best use of current antimicrobial effectiveness by reducing demand, e.g. through critical use of antibiotics, infection prevention and control, vaccination, provider and public education, and restriction to ‘last-resort’ antibiotics; and (iii) innovation to improve the efficacy of current therapy and develop new antimicrobial agents through research and development (R & D) schemes.

## Effective role of national public health institutes and surveillance systems

The COVID-19 pandemic also underscored the important role that effective national public health institutes and timely surveillance data play in the prevention and control of infectious diseases. Speakers emphasised that these institutions and activities play the same critical role for the AMR pandemic. In the case of AMR, this should extend to integrated One Health surveillance systems across human, animal and environmental domains to more effectively mitigate AMR. Speakers also addressed the importance of implementation research. Many of the evidence-based interventions addressing AMR have been based on data from high-income countries and such evidence cannot always be directly translated to low-resource settings. Thus, more (representative) data are needed in low-resource settings to tailor context-specific, cost-effective and sustainable interventions and facilitate their uptake into routine practice for the prevention and control of AMR in these settings. To carry out effective AMR surveillance and implementation research, critical gaps particularly affecting low-resource settings need to be addressed, such as lack of well-maintained laboratory equipment and consumables for bacteriology and quality control, trained personnel, reliable water, sanitation and hygiene systems in healthcare institutions, and information systems which can capture, manage and analyse data in a timely way. Generated surveillance data on AMR and AMC should be consequently used for political decision-making, infection prevention and control programmes as well as for advocacy.

## Conclusions

The discussions and conclusions on the five key themes in the meeting will be reflected in the new WHO CC at the RKI and fed by it into the ongoing planning and development of the WHO AMR Surveillance and Quality Assessment Collaborating Centres Network’s activities. More information on the Network and RKI’s role can be found under the following links:

- https://www.who.int/initiatives/glass/network
- https://www.rki.de/EN/Content/Institute/International/WHO_CC_EIBT/Coordinator-WHO-Antimicrobial-Resistance-Network.html
